# Supplementary material for: scFseCluster: a feature selection-enhanced clustering for single-cell RNA-seq data
Source: Life Sci Alliance. 2023 Oct 3;6(12):e202302103. doi: 10.26508/lsa.202302103 (PMC10547911; doi:10.26508/lsa.202302103)
Supplement: Supplementary file 5 [file LSA-2023-02103_TableS1.docx]

**Supplementary Table**

**Table S1**. The number of markers inferred by Seurat, and the overlaps with the Top 50 genes in FSQSSA’s replicates. The clusters and markers for each scRNA-seq dataset are predicted by Seurat. Top 50 genes are collected after repeating FSQSSA independently 500 times.

| **Dataset** | **No. of Cluster** | **No. of Marker gene** | **Overlapped with Top 50 genes** |
| --- | --- | --- | --- |
| Goolam | 4 | 4 | 4 |
| Darmanis | 5 | 5 | 5 |
| Usoskin | 8 | 8 | 8 |
| Xin | 9 | 9 | 8 |
| Romanov | 15 | 15 | 10 |
| 10X PBMC | 12 | 12 | 8 |
| Montoro | 14 | 14 | 11 |
| Hrvatin | 17 | 17 | 12 |
