# Supplementary material for: scFseCluster: a feature selection-enhanced clustering for single-cell RNA-seq data
Source: Life Sci Alliance. 2023 Oct 3;6(12):e202302103. doi: 10.26508/lsa.202302103 (PMC10547911; doi:10.26508/lsa.202302103)
Supplement: Supplementary file 6 [file LSA-2023-02103_TableS2.docx]

**Supplementary Table**

**Table S2.** The information of seven comparative methods for scRNA-seq clustering.

| **Methods** | **Type** | **Version** | **Journal** | **Year** |
| --- | --- | --- | --- | --- |
| Seurat ^(1)^ | Graph-based | 4.0.0 | Nature biotechnology | 2015 |
| scDeepCluster ^(2)^ | Autoencoder | 1.0.0 | Nature machine intelligence | 2019 |
| CIDR ^(3)^ | Hierarchical | 0.1.5 | Genome biology | 2017 |
| DESC ^(4)^ | Autoencoder | 2.1.1 | Nature communications | 2020 |
| SINCERA ^(5)^ | Hierarchical | 0.99.0 | PLoS computational biology | 2015 |
| SC3 ^(6)^ | K-means | 1.18.0 | Nature methods | 2017 |
| SIMLR ^(7)^ | Multi kernel learning | 1.24.0 | Nature methods | 2017 |

**References**

1. Satija, R., Farrell, J.A., Gennert, D., Schier, A.F. and Regev, A. (2015) Spatial reconstruction of single-cell gene expression data. *Nature biotechnology*, **33**, 495-502.

2. Tian, T., Wan, J., Song, Q. and Wei, Z. (2019) Clustering single-cell RNA-seq data with a model-based deep learning approach. *Nature Machine Intelligence*, **1**, 191-198.

3. Lin, P., Troup, M. and Ho, J.W. (2017) CIDR: Ultrafast and accurate clustering through imputation for single-cell RNA-seq data. *Genome biology*, **18**, 1-11.

4. Li, X., Wang, K., Lyu, Y., Pan, H., Zhang, J., Stambolian, D., Susztak, K., Reilly, M.P., Hu, G. and Li, M. (2020) Deep learning enables accurate clustering with batch effect removal in single-cell RNA-seq analysis. *Nature communications*, **11**, 1-14.

5. Guo, M., Wang, H., Potter, S.S., Whitsett, J.A. and Xu, Y. (2015) SINCERA: a pipeline for single-cell RNA-Seq profiling analysis. *PLoS computational biology*, **11**, e1004575.

6. Kiselev, V.Y., Kirschner, K., Schaub, M.T., Andrews, T., Yiu, A., Chandra, T., Natarajan, K.N., Reik, W., Barahona, M. and Green, A.R. (2017) SC3: consensus clustering of single-cell RNA-seq data. *Nature methods*, **14**, 483-486.

7. Wang, B., Zhu, J., Pierson, E., Ramazzotti, D. and Batzoglou, S. (2017) Visualization and analysis of single-cell RNA-seq data by kernel-based similarity learning. *Nature methods*, **14**, 414-416.
